# Double-Crosslinked H-PAN/MoS_2_/PEI Composite Nanofiltration Membrane for Ethanol Systems: Fabrication and Dye Separation Performance

**DOI:** 10.3390/membranes15100286

**Published:** 2025-09-23

**Authors:** Yixin Zhang, Chunli Liu, Lei Zhu, Xin Zhou, Miaona Wang, Yongqian Shen

**Affiliations:** 1 State Key Laboratory of Gansu Advanced Non-Ferrous Metal Materials, Lanzhou University of Technology, Lanzhou 730050, China; 2 School of Material Science and Engineering, Lanzhou University of Technology, Lanzhou 730050, China

**Keywords:** organic solvent nanofiltration, MoS_2_ nanosheets, thermal-crosslinking, quaternization-crosslinking, dye separation

## Abstract

Organic solvent nanofiltration (OSN) is a promising technology for solute removal from organic media, yet developing membranes with stable separation performance remains challenging. This study presents a solvent-resistant double-crosslinked nanofiltration membrane fabricated via a two-step strategy: preparation of the membrane by the polyion complexion reaction-assisted non-solvent-induced phase inversion (PIC-assisted NIPS) method and then post-crosslinking with hydrothermal treatment followed by quaternization with 1,3,5-tris(bromomethyl)benzene (TBB). To enhance solvent stability, molybdenum sulfide (MoS_2_) nanosheets were incorporated into a hydrolyzed polyacrylonitrile (H-PAN) substrate. The H-PAN/MoS_2_/PEI base membrane was fabricated by PIC-assisted NIPS with a polyethylenimine (PEI) aqueous solution as the coagulation bath. The membrane subsequently underwent dual crosslinking comprising hydrothermal treatment and 1,3,5-tris(bromomethyl)benzene (TBB)-mediated quaternization crosslinking, ultimately yielding the H-PAN/MoS_2_/PEI (Ther.+TBB QCL) composite membrane. These crosslinking procedures reduced the membrane’s separation skin layer thickness from 64 nm (uncrosslinked) to 41 nm. The resultant membrane effectively separated dyes from ethanol, achieving a rejection rate of 97.0 ± 0.9% for anionic dyes (e.g., Congo Red) and a permeance flux of 23.6 ± 0.2 L·m^−2^·h^−1^·bar^−1^ at 0.4 MPa. Furthermore, after 30 days of immersion in ethanol at 25 °C, its flux decay rate was markedly lower than that of a non-crosslinked control membrane. The enhanced separation performance and stability are attributed to the thermal crosslinking promoting amide bond formation and the TBB crosslinking introducing quaternary ammonium groups. This double-crosslinking strategy offers a promising approach for preparing high-performance OSN membranes.

## 1. Introduction

Organic solvents are widely used in the chemical and pharmaceutical industries [1,2], where their separation and purification are critical for environmental protection and cost efficiency [3,4]. As a membrane-based separation technology, organic solvent nanofiltration (OSN) is recognized as an eco-friendly and energy-efficient approach [5,6]. Consequently, nanofiltration membranes with exceptional and stable performance in organic media have attracted significant attention due to their high solvent resistance and consistent separation performance [7,8,9,10].

Integrally skinned asymmetric (ISA) membranes fabricated through non-solvent-induced phase separation (NIPS) have become prevalent in organic solvent nanofiltration (OSN) applications owing to their characteristic thin selective layer/porous support layer architecture [11,12]. A critical limitation of conventional NIPS-prepared polymeric membranes lies in their inherent swelling behavior when exposed to casting solution solvents, necessitating enhanced solvent stability for practical OSN implementation. Among various stabilization approaches, crosslinking techniques, particularly ultraviolet-induced, thermal, and chemical crosslinking, have proven most effective for improving membrane solvent resistance [13,14]. Thermal crosslinking has garnered particular research interest due to its operational simplicity and processing efficiency [15,16,17,18], despite its well-documented trade-off between enhanced solvent stability and undesirable pore structure collapse/melting that invariably compromises membrane permeance [19,20,21,22]. This dichotomy has driven current investigations into maintaining membrane permeability while preventing thermal-induced pore degradation, with promising results achieved through strategic incorporation of rigid 1D/2D nanostructured reinforcements that enhance mechanical integrity and minimize high-temperature pore deformation. Recent advances in 2D nanomaterial-engineered membranes demonstrate exceptional structural integrity under harsh processing conditions. A variety of two-dimensional (2D) nanomaterials have been extensively explored as advanced nanofillers in thin-film nanocomposite (TFN) membranes for organic solvent nanofiltration (OSN), owing to their unique structural and physicochemical properties. Graphene oxide (GO) nanosheets, for instance, are widely incorporated due to their abundant oxygen-containing functional groups and tunable interlayer spacing, which facilitate enhanced solvent permeance and selective molecular transport [23,24]. Similarly, MXene nanosheets—characterized by their metallic conductivity and hydrophilic surface terminals—have demonstrated exceptional potential in constructing high-flux and antifouling OSN membranes [25,26]. Very recently, Xue et al. engineered surface-porous MXene (SPMXene) nanosheets via a synergistic etching strategy, which endowed TFN membranes with multi-channel water transport pathways and significantly improved permeance without compromising rejection [27]. Wang et al. developed braid-reinforced hollow fiber membranes exhibiting mechanical robustness in organic solvents, though intrinsic polymer instability persists during thermal crosslinking [28]. Crucially, covalent modification strategies for MoS_2_ nanosheets prove effective in suppressing membrane swelling and structural collapse during solvent exposure, as demonstrated by Wang et al. in thin-film composites [29]. This stability enhancement aligns with molecular dynamics simulations by Liu et al., revealing that 2D MXene interlayer channels maintain dimensional confinement under chemical stress, resisting pore deformation through nanoconfinement effects [30]. Further mechanistic insight arises from Wu et al.’s work, where SWCNT-channeled MOF nanosheets create rigid percolation pathways that decouple solvent transport from matrix contraction [31]. Collectively, these studies establish that 2D nanomaterials act as structural scaffolds, mitigating pore collapse during post-treatment—a principle central to our design of thermally stabilized H-PAN/MoS_2_ membranes. Covalent organic framework (COF) nanosheets also emerge as promising candidates due to their highly ordered pore structures and designable functionality, enabling precise molecular sieving in organic environments [32,33]. Despite these advancements, challenges remain in achieving uniform dispersion, avoiding interfacial defects, and maintaining long-term stability under harsh solvent conditions. In this context, MoS_2_ nanosheets offer distinct advantages, including robust mechanical properties, ease of functionalization, and high chemical stability, making them particularly suitable for constructing solvent-resistant nanofiltration membranes [34,35]. Our work builds upon these foundations by incorporating MoS_2_ into a double-crosslinked H-PAN matrix to address the critical issue of membrane swelling and performance decay in ethanol systems.

Furthermore, the quaternary ammonium crosslinking (QCL) strategy demonstrates significant potential for enhancing membrane structural stability and selective separation performance primarily through the formation of a stable and rigid covalent network. This crosslinking mechanism effectively restricts polymer chain mobility to mitigate swelling-induced pore deformation and compaction under pressure, thereby well-maintaining the precise size-sieving properties of the membrane in organic solvents [36,37]. Our previous development of H-PAN-based tight ultrafiltration membranes via phase inversion demonstrated this principle, where in situ polyelectrolyte complexation yielded nanoscale-thick selective layers with exceptional dye removal efficiency in aqueous systems. However, this study primarily focused on aqueous separation systems, resulting in inadequate solvent resistance in polar organic solvent environments. Consequently, it becomes crucial to develop membranes that maintain high permeance while possessing ultrathin, solvent-resistant selective layers capable of withstanding harsh solvent conditions.

This study presents an innovative dual-crosslinking strategy integrated with ultrathin selective layer engineering to advance H-PAN-based nanofiltration membranes for organic solvent applications. Building upon our previous work [38], where an ultrathin selective skin layer was initially fabricated during membrane formation, we addressed the critical challenge of solvent-induced swelling and performance deterioration in organic media (particularly ethanol) by incorporating rigid two-dimensional MoS_2_ nanosheets into the membrane matrix. This structural modification effectively mitigated pore collapse and size reduction during thermal treatment. Through sequential crosslinking involving (1) hydrothermal treatment and (2) 1,3,5-tris(bromomethyl)benzene (TBB)-mediated quaternization crosslinking, we successfully fabricated dual-crosslinked H-PAN/MoS_2_/PEI (Ther.+TBB QCL) membranes featuring an optimized ultrathin selective layer. This synergistic design combining ultrathin layer architecture with dual crosslinking not only significantly enhanced ethanol resistance but also resolved the traditional trade-off between high permeance and solvent stability, thereby providing an innovative membrane solution for efficient dye separation in ethanol systems.

## 2. Materials and Methods

### 2.1. Materials

**Figure A1:**
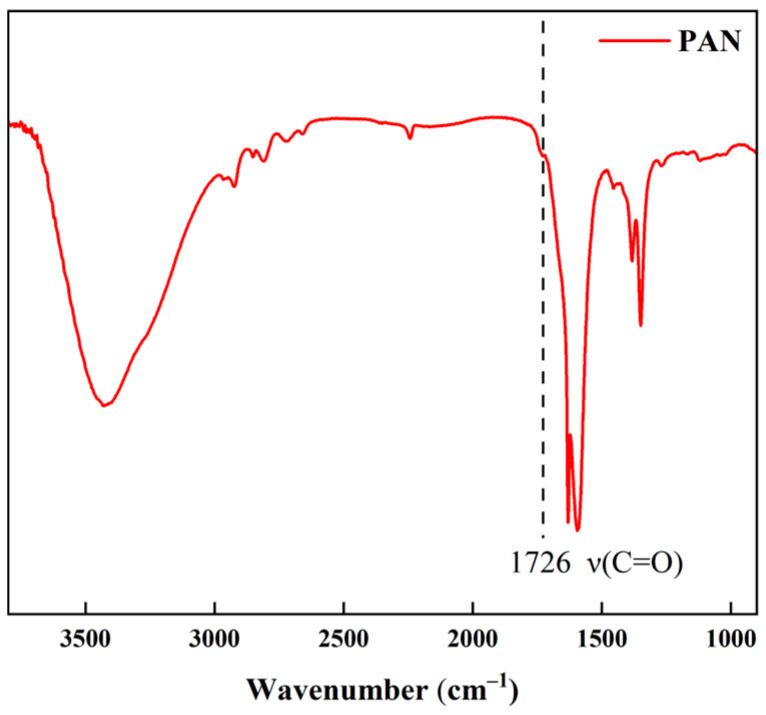
FTIR spectrum of the polyacrylonitrile (PAN) material (Mw = 80,000) used in this study.

Polyacrylonitrile (PAN, Mw = 80,000 Da, with the IR spectrum shown in Figure A1, Appendix B) and sodium hydroxide were purchased from Shanghai Macklin Biochemical Technology, Shanghai, China. N-Methyl-2-pyrrolidone (NMP) was obtained from Tianjin Guangfu Technology Development, Tianjin, China. Polyvinylpyrrolidone (PVP K30) was sourced from Tianjin Zhiyuan Chemical Reagent, Tianjin, China. Anhydrous ethanol was acquired from Tianjin Baishi Chemical Reagent, Tianjin, China. Dyes were supplied as follows: Sunset Yellow (azo) from Tianjin Kaixin Chemical Industry, Tianjin, China; Direct Red (Yuanye Co., Shanghai, China); Congo Red (azo, Tianjin Tianxin Fine Chemical, Tianjin, China); Reactive Black (TCI Shanghai Co., Shanghai, China); Evans Blue (Beijing Innochem, Beijing, China). Additional materials from Beijing Innochem included: MoS_2_ nanosheets (>99% metals basis, nanopowder, 90 nm diameter), polyethylenimine (PEI, Mw = 10,000), and 1,3,5-tris(bromomethyl)benzene (TBB). Laboratory-produced deionized water was used. All chemicals were analytical grade and employed without purification.

### 2.2. Preparation of the H-PAN/MoS_2_/PEI (Ther.+TBB QCL) Membrane

The H-PAN was prepared according to our previously reported method [38]. Specifically, 6.4 g of pre-synthesized hydrolyzed polyacrylonitrile (H-PAN) and 2.0 g of polyvinylpyrrolidone K30 (PVP K30) were dissolved in a measured amount of N-methyl-2-pyrrolidone (NMP) under mechanical stirring at 80 °C. Simultaneously, 0.012 g of molybdenum sulfide (MoS_2_) nanosheets were dispersed in a separate portion of NMP. The MoS_2_ dispersion was then incorporated into the H-PAN solution, and the mixture was continuously stirred for 30 min to ensure complete homogenization, resulting in a homogeneous and transparent mixture with a polymer solid content of 16.0 wt% and MoS_2_ content of 0.03 wt%. The total amount of NMP used was 31.6 g. This mixed solution was centrifuged at 8000 rpm for 15 min to remove air bubbles and impurities, followed by aging at 30 °C for 12 h to obtain the final casting solution. The viscosity of the resulting casting solution was measured at room temperature (24 ± 0.5 °C) using a rotational viscometer (DV2T, BROOKFIELD AMETEK, Middleborough, MA, USA). The reported value, 8663.0 ± 42.8 mPa·s, represents the average of five independent measurements.

**Figure 1:**
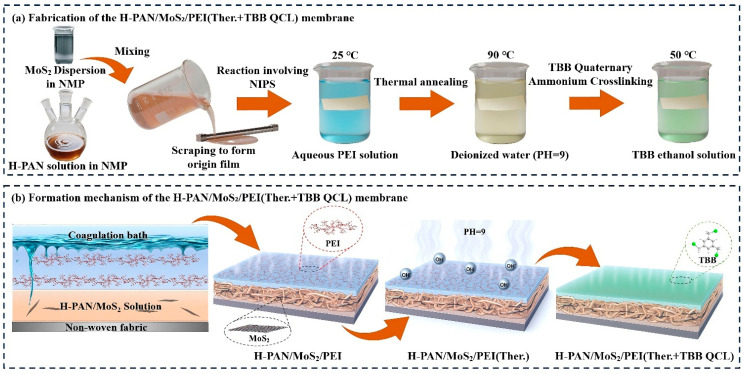
Schematic illustration of (**a**) the fabrication process and (**b**) the formation mechanism of the H-PAN/MoS_2_/PEI (Ther.+TBB QCL) membrane.

Coagulation baths comprised deionized water and aqueous polyethylenimine (PEI) solutions (0.2 wt%, 0.4 wt%, 0.6 wt%, 0.8 wt%, 1.0 wt%). Membrane fabrication at 25 °C involved casting the dope solution onto non-woven fabric using a 150-μm doctor blade. The nascent membrane was immediately immersed in the coagulation bath for non-solvent induced phase separation (NIPS), followed by thorough water washing to eliminate residual solvents. The as-prepared H-PAN/MoS_2_/PEI membrane was first subjected to thermal crosslinking in deionized water (pH = 9) at 90 °C for 3 h. Following thorough rinsing with deionized water, the resulting H-PAN/MoS_2_/PEI (Ther.) membrane was obtained. After carefully removing surface moisture by blot drying, the membrane was immersed in a 0.1 wt% 1,3,5-tris(bromomethyl)benzene (TBB) ethanol solution at 50 °C for 6 h to complete the quaternization crosslinking process. The final dual-crosslinked H-PAN/MoS_2_/PEI (Ther.+TBB QCL) membrane was stored in ethanol for subsequent characterization. The fabrication process and membrane formation mechanism for the H-PAN/MoS_2_/PEI (Ther.+TBB QCL) membrane is depicted in Figure 1.

### 2.3. Membrane Characterization

The membrane’s cross-sectional morphology and selective skin layer thickness were characterized using scanning electron microscopy (SEM, CHINAINSTRU & QUANTUMTECH, Hefei, China). Prior to imaging, the membrane samples were cryogenically fractured in liquid nitrogen and sputter-coated with a thin gold layer to enhance conductivity and prevent charging effects. Simultaneously, the surface elemental composition and distribution were analyzed through energy-dispersive X-ray spectroscopy (EDS) coupled with SEM. The surface topography and roughness parameters of the membrane were examined using atomic force microscopy (AFM, CSPM5500, Benyuan Nano Instrument Co., Ltd., Guangzhou, China). The chemical structure of the membrane surface was examined by attenuated total reflectance Fourier-transform infrared spectroscopy (ATR-FTIR, Nexus 670, Thermo Nicolet Corporation, Waltham, MA, USA), and the spectra were collected in the range of 4000–500 cm^−1^ with a resolution of 4 cm^−1^ and 32 accumulated scans. While the X-ray photoelectron spectroscopy (XPS, Shimadzu/Kratos Axis Supra, Kiyamachi, Japan) was employed to determine the surface elemental composition and chemical bonding states. Surface wettability was evaluated via water contact angle (WCA) measurements using the sessile drop method on an OCA20 instrument (DataPhysics, Filderstadt, Germany) at ambient temperature, with reported values representing the average of at least five measurements taken at different locations on the membrane surface.

### 2.4. Organic Solvent Nanofiltration Performance Testing

Membrane permeance was evaluated using a cross-flow filtration system with three cells (effective area: 21.2 cm^2^). Membranes were pre-compacted with ethanol at 0.4 MPa for 60 min to achieve physicochemical equilibrium. Following flux stabilization, ethanol permeance was recorded. Separation performance was subsequently assessed using dye-containing ethanol solutions with dye concentration of 0.05 g·L^−1^, measuring both permeance flux and dye rejection. Ethanol Flux (*P*, L·m^−2^·h^−1^), Permeance (*P*, L·m^−2^·h^−1^·bar^−1^) and dye rejection rate (*R*, %) were calculated using the following Equations (1)–(3):

(1) $$F=\frac{V}{A\times t\;}$$

(2) $$P=\frac{F}{\Delta P\;}$$

where *V* is permeating volume (L), *t* is time (h), and *A* is effective membrane area (cm^2^), Δ*P* is the operation pressure (bar).

(3) $$R=(1-\frac{C_{P}}{C_{F}})\times 100\%$$

where *C_P_* and *C_F_* represent dye concentrations in permeate and feed solutions, respectively. Concentrations were quantified using a UV-vis spectrophotometer (Model 752 N, Shanghai Yidian Analytical Instruments, Shanghai, China) at each dye’s maximum absorption wavelength.

### 2.5. Membrane Antifouling Performance Evaluation

The antifouling performance of H-PAN/MoS_2_/PEI (Ther.+TBB QCL) membranes was evaluated using humic acid (HA) as a model pollutant. Prior to fouling tests, membranes were pre-compacted with ethanol at 0.4 MPa for 1.5 h to establish initial permeance flux (*J_w_*_1_). The feed was then replaced with 0.1 g·L^−1^ HA in ethanol, and permeate flux (*J_p_*) was recorded. Normalized flux ratio (*J_p_*/*J_w_*_1_) monitored flux decline behavior during constant-pressure operation. Post-fouling, membranes underwent cleaning for flux recovery assessment: ethanol rinsing at 0.4 MPa for 1.5 h with intermittent solvent replacement ensured thorough cleaning, followed by measurement of restored permeance flux (*J_w_*_2_). Antifouling performance was quantified through flux recovery ratio (*FRR*), total flux decline ratio (*DR_t_*), reversible flux declines ratio (*DR_r_*), and irreversible flux decline ratio (*DR_ir_*), calculated as follows Equations (4)–(7) [39]:

(4) $$FRR=\frac{J_{w2}}{J_{w1}}\times 100\%$$

(5) $${DR}_{t}=\left(1-\frac{J_{p}}{J_{w1}}\right)\times 100\%$$

(6) $${DR}_{r}=\left(\frac{J_{w2}-J_{p}}{J_{w1}}\right)\times 100\%$$

(7) $${DR}_{ir}=\left(1-\frac{J_{w2}}{J_{w1}}\right)\times 100\%$$

### 2.6. Solvent Stability Evaluation

Membrane samples were immersed in ethanol at 25 °C for 30 days. Post immersion, samples were briefly ethanol-rinsed to remove surface residues. Following Section 2.4 protocols (0.4 MPa, 25 °C, 0.05 g·L^−1^ Congo Red in ethanol), post-immersion ethanol flux (*F_t_*), permeate flux (*F*_0_) and Congo Red rejection (*R_t_*) were measured. *DR_F_* and *DR_R_* were calculated by comparing (*F_t_*, *R_t_*) with pre-immersion baselines (*F*_0_, *R*_0_). Lower *DR* values indicate superior long-term stability under solvent exposure.

Membrane solvent resistance performance was evaluated by measuring the ethanol flux, dye permeate flux of ethanol, and dye rejection before and after 30-day ethanol exposure at 25 °C, with flux decay rate (*DR_F_*) calculated according to Equation (8):

(8) $${DR}_{F}=\left(\frac{F_{0}-F_{t}}{F_{0}}\right)\times 100\%$$

where *F*_0_ is the initial ethanol flux or dye permeate flux of ethanol, and *F_t_* is the value after 30-day immersion. The rejection decay rate (*DR_R_*) was calculated using Equation (9):

(9) $${DR}_{R}=\left(\frac{R_{0}-R_{t}}{R_{0}}\right)\times 100\%$$

where *R*_0_ is the initial dye rejection (%), and *R_t_* is the rejection after 30 days.

## 3. Results

**Figure 2:**
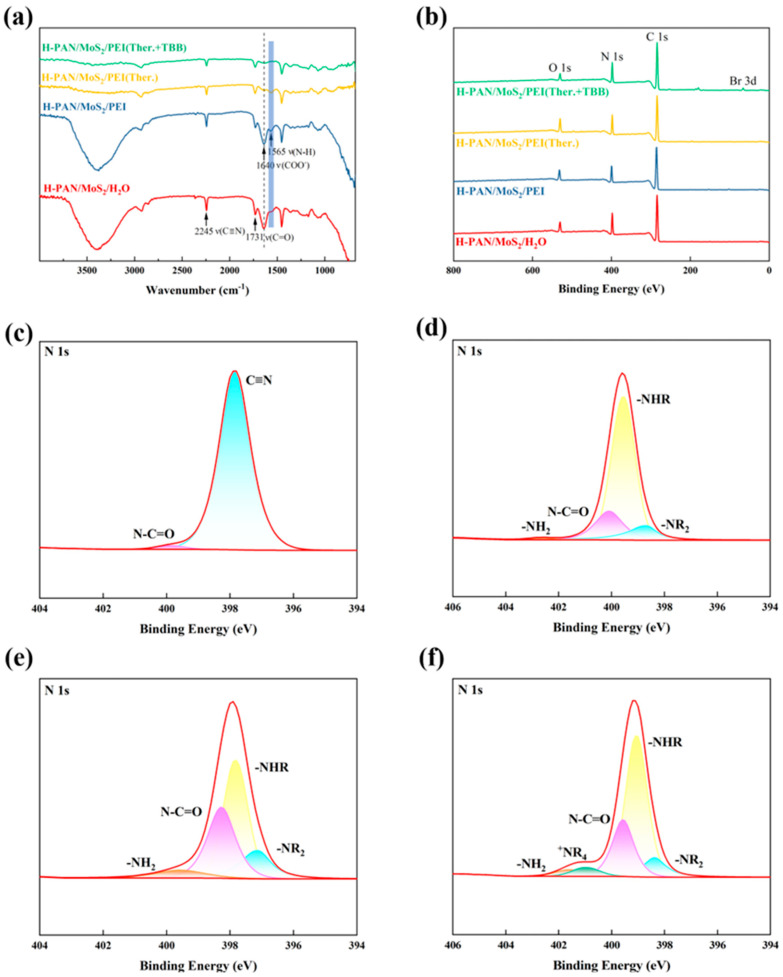
Characterization of composite membranes: (**a**) FTIR spectra and (**b**) XPS survey spectra of H-PAN/MoS_2_, PAN/MoS_2_/PEI, PAN/MoS_2_/PEI (Ther.), and H-PAN/MoS_2_/PEI (Ther.+TBB QCL) membranes; (**c**–**f**) are the high-resolution N1s XPS spectra corresponding to the above membranes, respectively.

The chemical composition of the H-PAN/MoS_2_, H-PAN/MoS_2_/PEI, H-PAN/MoS_2_/PEI (Ther.), and the H-PAN/MoS_2_/PEI (Ther.+TBB QCL) membrane surface was characterized by FTIR, as presented in Figure 2a. The H-PAN/MoS_2_ membrane exhibits a characteristic carboxyl (−COOH) C=O stretching vibration at 1731 cm^−1^, originating from cyanide group hydrolysis in hydrolyzed polyacrylonitrile, along with a broad and strong O–H stretching vibration band in the 3000–3600 cm^−1^ region also arising from the hydroxyl groups of carboxyl functionalities. H-PAN/MoS_2_/PEI membrane displays an N−H bending vibration at 1565 cm^−1^, confirming successful PEI-derived amino group incorporation, while the absorption in the 3000–3600 cm^−1^ region slightly broadens due to overlapping N–H stretching vibrations from primary and secondary amines in PEI. For H-PAN/MoS_2_/PEI (Ther.) membrane, significant attenuation of the 1565 cm^−1^ amino peak versus its precursor and 1640 cm^−1^ of the COO^−^ groups indicates carboxyl-amino reactions under heated alkaline conditions that deplete the content of amino groups on membrane surface; concurrently, the intensity of the O–H/N–H absorption band in the 3000–3600 cm^−1^ region decreases and broadens further, suggesting consumption of hydroxyl and amino groups via amination. Moreover, the gradual decrease in the intensity of the characteristic peak at 2245 cm^−1^, assigned to the stretching vibration of unhydrolyzed nitrile groups (–C≡N) in PAN, from H-PAN/MoS_2_/PEI to the crosslinked membranes indicates further hydrolysis of residual cyano groups into carboxyl groups under weakly alkaline conditions during thermal treatment, followed by participation in amide bond formation. The H-PAN/MoS_2_/PEI (Ther.+TBB QCL) spectrum reveals a C−N stretching vibration at 1060 cm^−1^, confirming amide bond formation between residual PEI amines and 1,3,5-tris(bromomethyl)benzene [40], while further reduction in the 1640 cm^−1^ peak evidences quaternary ammonium salt formation via the quaternization of amino groups.

XPS survey spectra and N1s high-resolution spectra of H-PAN/MoS_2_, H-PAN/MoS_2_/PEI, H-PAN/MoS_2_/PEI (Ther.), and H-PAN/MoS_2_/PEI (Ther.+TBB QCL) membranes are presented in Figure 2b–f. The survey spectra (Figure 2b) reveal dominant C, N, and O elements in H-PAN/MoS_2_/PEI (Ther.), while the H-PAN/MoS_2_/PEI (Ther.+TBB QCL) membrane exhibits a new Br 3d peak confirming bromine incorporation in the post-TBB crosslinking reaction. Deconvoluted N1s spectra (Figure 2c–f) demonstrate progressive chemical modifications: H-PAN/MoS_2_ exhibits characteristic peaks at 398.6 eV (–C≡N) and 399.5 eV (–N–C=O); H-PAN/MoS_2_/PEI features additional components at 398.6 eV (–NR_2_), 399.1 eV (–NHR), and 401.5 eV (–NH_2_) alongside 399.5 eV (–N–C=O), indicating PEI complexation via polyelectrolyte interactions during phase inversion; H-PAN/MoS_2_/PEI (Ther.) retains these signatures with significantly intensified –N–C=O (399.5 eV) peak, demonstrating carboxyl-amino amination under weakly alkaline conditions [17]; finally, H-PAN/MoS_2_/PEI (Ther.+TBB QCL) shows a distinct ^+^NR_4_ peak at 400.8 eV concurrent with residual peaks, confirming TBB-PEI quaternization [40].

**Figure 3:**
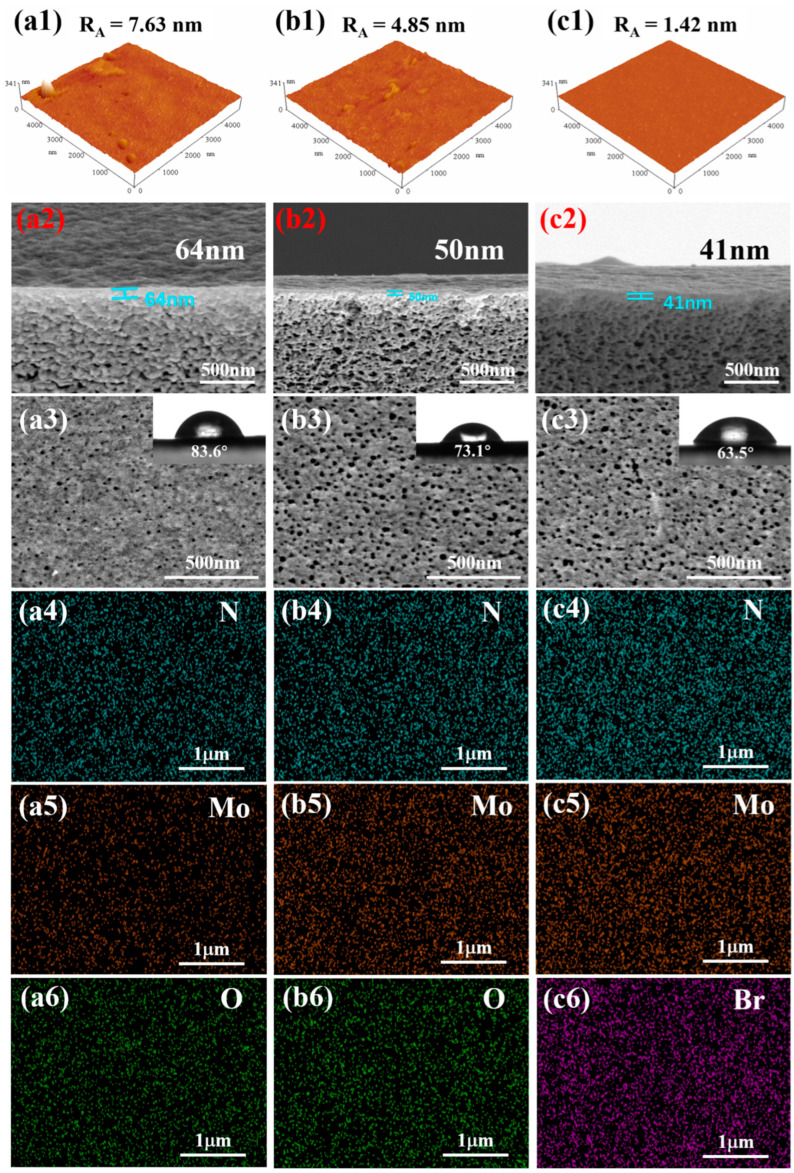
The AFM images, Surface and cross-sectional morphology of the membranes: (**a1**–**a3**) the H-PAN/MoS_2_/PEI membrane, (**b1**–**b3**) H-PAN/MoS_2_/PEI (Ther.) membrane, and (**c1**–**c3**) H-PAN/MoS_2_/PEI (Ther.+TBB QCL) membrane (inset: the corresponding surface roughness values and WCA of membrane surface); The EDS analysis of surface elements: (**a4**) N, (**a5**) Mo, and (**a6**) O for H-PAN/MoS_2_/PEI membrane; (**b4**) N, (**b5**) Mo, and (**b6**) O for H-PAN/MoS_2_/PEI (Ther.) membrane; (**c4**) N, (**c5**) Mo, and (**c6**) Br for H-PAN/MoS_2_/PEI (Ther.+TBB QCL) membrane.

Atomic force microscopy (AFM) was employed to quantitatively analyze the surface topography and roughness of the membranes, as shown in Figure 3(a1–c1). The measured root mean square roughness (R_A_) values decreased progressively from 7.63 nm for the H-PAN/MoS_2_/PEI membrane, to 4.85 nm for the H-PAN/MoS_2_/PEI (Ther.) membrane, and finally to 1.42 nm for the H-PAN/MoS_2_/PEI (Ther.+TBB QCL) membrane. This consistent reduction in surface roughness quantitatively confirms that each crosslinking step effectively promotes the formation of a smoother and more homogeneous membrane surface. Cross-sectional and surface morphology of H-PAN/MoS_2_/PEI, H-PAN/MoS_2_/PEI (Ther.), and H-PAN/MoS_2_/PEI (Ther.+TBB QCL) membranes was characterized by SEM, as shown in Figure 3(a2–c2). Cross-sectional morphology analysis revealed progressive thinning of the separation skin layer with increasing crosslinking density: thermal crosslinking reduces skin thickness from 64 nm in H-PAN/MoS_2_/PEI to 50 nm in H-PAN/MoS_2_/PEI (Ther.), while subsequent TBB crosslinking further decreases it to 41 nm in H-PAN/MoS_2_/PEI (Ther.+TBB QCL). The transition layer exhibits abundant micro-pores. Surface SEM images acquired in dry state do not accurately represent pore dimensions under operational wet conditions due to slight swelling induced by polymer-ethanol interactions during filtration.

Water contact angle (WCA) measurements reflect both surface ionic group content and membrane affinity toward polar solvents. The WCAs of the four membranes reveal progressive enhancement in hydrophilicity after the thermal and quaternization crosslinking treatment of membrane: H-PAN/MoS_2_/PEI exhibits a WCA of 83.6°, while thermal crosslinking reduces this to 73.1° for H-PAN/MoS_2_/PEI (Ther.), indicating enhanced wetting by polar solvents and improved solvent affinity. The H-PAN/MoS_2_/PEI (Ther.+TBB QCL) membrane shows a further reduced WCA of 63.5°, demonstrating that quaternization crosslinking introduces additional polar groups, thereby strengthening polar solvent interactions beyond the thermally crosslinked counterpart.

**Figure 4:**
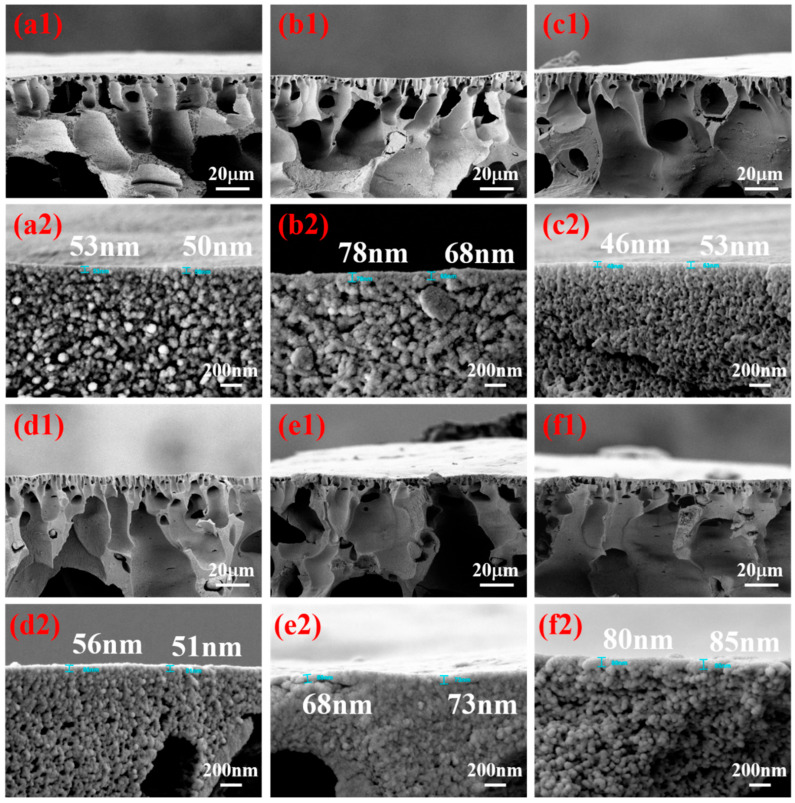
(**a1**–**f1**) Cross-sectional morphology of H-PAN/MoS_2_/PEI (Ther.+TBB QCL) membranes prepared with different PEI concentrations in the coagulation bath (0 wt%, 0.2 wt%, 0.4 wt%, 0.6 wt%, 0.8 wt%, and 1.0 wt%), showing the overall membrane structure; (**a2**–**f2**) Magnified views of the selective skin layer region of the corresponding membranes.

**Figure 5:**
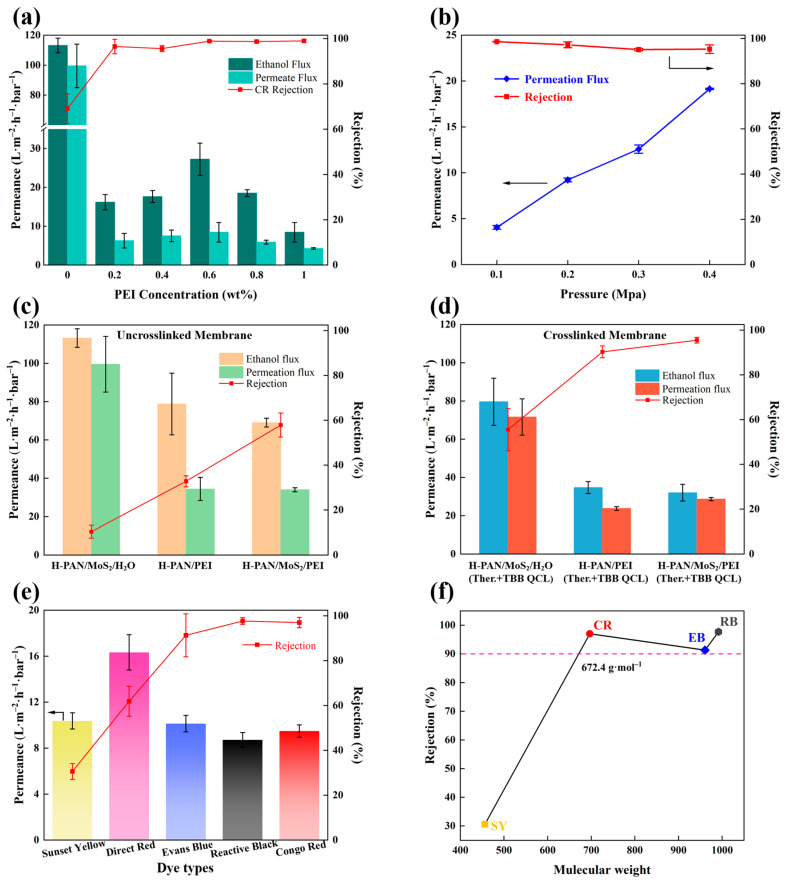
(**a**) Flux and rejection rate of H-PAN/MoS_2_/PEI (Ther.+TBB QCL) membrane with different PEI concentrations; (**b**) Flux and rejection rate under different pressures; The flux and rejection of three membranes without crosslinking (**c**) and after dual crosslinking (**d**); (**e**) The performance for separation of different dyes in ethanol solution and (**f**) MWCO of the H-PAN/MoS_2_/PEI (Ther.+TBB QCL) membrane (OSN conditions: 0.4 MPa, 0.05 g·L^−1^ dye/ethanol solution).

Figure 4 presents the cross-sectional morphology of H-PAN/MoS_2_/PEI (Ther.+TBB QCL) membranes prepared with different polyethylenimine (PEI) concentrations in the coagulation bath. The low-magnification images (a1–f1) reveal that all membranes exhibit a typical asymmetric structure, consisting of a dense selective skin layer and a macrovoids-rich support layer. Close examination of the selective skin layer in the high-magnification images (a2–f2) shows that the skin layer is relatively thin at 0 wt% PEI. With the addition of 0.2 wt% PEI, the thickness increases slightly, while a further increase to the optimum concentration of 0.6 wt% results in a substantial reduction to the minimum thickness. However, when the PEI concentration is increased beyond this point to 0.8 wt% and 1.0 wt%, this trend reverses, and the skin layer becomes thicker again. This morphological evolution correlates well with the separation performance shown in Figure 5a, where the highest flux and rejection are achieved at 0.6 wt% PEI, confirming that the skin layer thickness is a key structural parameter determining the membrane’s separation performance. This trend can be attributed to the interplay of two mechanisms during membrane formation. At low PEI concentration (0.2 wt%), the initial increase in thickness results from rapid complexation between PEI and the H-PAN matrix at the interface, leading to localized polymer aggregation before a uniform skin layer is formed. When the PEI concentration reaches 0.6 wt%, the polyelectrolyte complexation becomes sufficient and uniform, promoting the formation of a denser and thinner selective layer. Beyond the optimum concentration (0.6 wt%), the increased viscosity of the coagulation bath becomes the dominant factor, slowing the interdiffusion between solvent and non-solvent and thereby delaying phase separation kinetics and reducing complexation efficiency. This ultimately leads to the formation of a thicker skin layer. Thus, the membrane prepared with 0.6 wt% PEI in the coagulation bath exhibits the thinnest skin layer, which is a critical factor contributing to its superior separation performance.

The effect of polyethyleneimine (PEI) concentration in the coagulation bath on the separation performance of H-PAN/MoS_2_/PEI (Ther.+TBB QCL) membranes was systematically investigated, with the results presented in Figure 5a. The membrane formed in a PEI-free coagulation bath exhibited relatively high pure ethanol flux (113.2 ± 4.9 L·m^−2^·h^−1^·bar^−1^) and ethanol permeation flux (99.5 ± 14.5 L·m^−2^·h^−1^·bar^−1^), but a low Congo red rejection rate of only 69.1 ± 6.5%. This indicates that without PEI-provided reaction sites, the double-crosslinking process fails to form a dense selective skin layer with efficient sieving capability. Introducing PEI into the coagulation bath rapidly increased Congo red rejection to above 90%, attributable to PEI complexing onto the membrane surface via polyion complexion reaction during phase inversion. Its abundant amino groups then serve as reaction sites for subsequent thermal crosslinking and TBB quaternization, forming a structurally dense and stable separation skin layer. Ethanol flux displayed an initial increase followed by a decrease with rising PEI concentration: within the 0.2–0.6 wt% range, ethanol flux increased from 16.2 ± 2 L·m^−2^·h^−1^·bar^−1^ to a peak of 24.3 ± 0.1 L·m^−2^·h^−1^·bar^−1^ at 0.6 wt%, but declined to 8.4 ± 2.5 L·m^−2^·h^−1^·bar^−1^ at 1.0 wt%. PEI concentration of 0.6 wt% in the coagulation bath was found to be optimal, providing adequate amino groups for effective dual-crosslinking while maintaining membrane performance. This concentration enabled the formation of a dense yet stable selective skin layer without causing the over-crosslinking and excessive densification observed at higher PEI concentrations. Based on this performance profile, 0.6 wt% PEI was selected as the optimal coagulation bath concentration.

Operating pressure significantly influences membrane separation performance, affecting both flux and rejection. Figure 5b shows the ethanol permeation flux and Congo red rejection of the H-PAN/MoS_2_/PEI (Ther.+TBB QCL) membrane at different operating pressures. The membrane demonstrated an ethanol permeation flux of 4.1 ± 0.2 L·m^−2^·h^−1^·bar^−1^ at 0.1 MPa, increasing to 19.2 ± 0.1 L·m^−2^·h^−1^·bar^−1^ at 0.4 MPa, indicating a approximately linear increase in permeation flux with rising pressure. This demonstrates that pressure is the primary driver of solvent permeation and confirms the absence of significant structural collapse within the 0.1–0.4 MPa range. Concurrently, Congo red rejection consistently exceeded 95% across this pressure range. This rejection insensitivity to pressure confirms the membrane’s exceptional size-sieving stability. Even at 0.4 MPa, no selectivity decline occurred due to compaction or excessive polymer chain swelling. The robust stability of the H-PAN/MoS_2_/PEI (Ther.+TBB QCL) membrane results from the dense, anti-swelling separation skin layer formed by thermal and TBB quaternization crosslinking, and the reinforcement provided by MoS_2_ nanosheets to the substrate.

Figure 5c,d are the performance of the uncrosslinked and crosslinked membranes, respectively. A comparative analysis of Figure 5c,d reveals that the membrane prepared without MoS_2_ in the casting solution experienced a more pronounced decline in permeance and had a relatively lower rejection after dual crosslinking. This behavior demonstrates that the incorporation of rigid two-dimensional MoS_2_ nanosheets effectively suppress excessive contraction of the polymer network during crosslinking, thereby preserving water transport pathways and mitigating flux reduction; Meanwhile, the membrane coagulated with PEI-containing bath exhibited a more significant improvement in rejection rate. This behavior can be attributed to the synergistic role of MoS_2_ nanosheets and PEI in the membrane structure. PEI significantly enhances crosslinking density through its abundant amino groups, leading to a more compact and stable selective layer with improved solute rejection. Furthermore, the MoS_2_ nanosheets in the casting solution tend to align parallel to the membrane plane under the shear force applied during the doctor-blading process. This oriented structure can also lead to an enhanced retention performance of the membrane. Consequently, the cooperative incorporation of MoS_2_ and PEI successfully enables the dual-crosslinked membrane to maintain high permeance while significantly enhancing separation selectivity. A comparison of the membrane performance with recently reported counterparts is provided in Table 1. The H–PAN/MoS_2_/PEI (Ther.+TBB QCL) membrane fabricated in this work exhibits relatively higher permeability and dye rejection rates.

As shown in Figure 5e, the H-PAN/MoS_2_/PEI (Ther.+TBB QCL) membrane effectively separates various dyes from ethanol, including Sunset Yellow (SY, 452.4 g/mol), Direct Red (DR, 704.7 g/mol), Evans Blue (EB, 960.8 g/mol), Reactive Black (RB, 991.8 g/mol), and Congo Red (CR, 696.7 g/mol). The properties of the organic dyes are presented in Table A1 (Appendix A). Figure 5f indicates that the membrane exhibits high rejections for RB, CR, and EB, with a molecular weight cut-off (MWCO) of 672.4 g/mol at 90% rejection. In membrane separation, solute rejection is governed primarily by the hydrodynamic diameter (effective size) rather than the nominal molecular weight. In ethanol, dye aggregation behavior significantly influences effective molecular dimensions. CR, with its rigid binaphthyl structure, tends to form large aggregates through π-π stacking in the low-dielectric-constant environment, leading to an effective size much larger than that expected from its monomeric molecular weight. In contrast, although EB has a higher molecular weight, its multiple polar sulfonate groups suppress extensive aggregation, resulting in the prevalence of monomers or smaller aggregates. Thus, the larger effective size of CR aggregates corresponds to its higher rejection (97.0%) compared to EB (91.3%).

**Figure 6:**
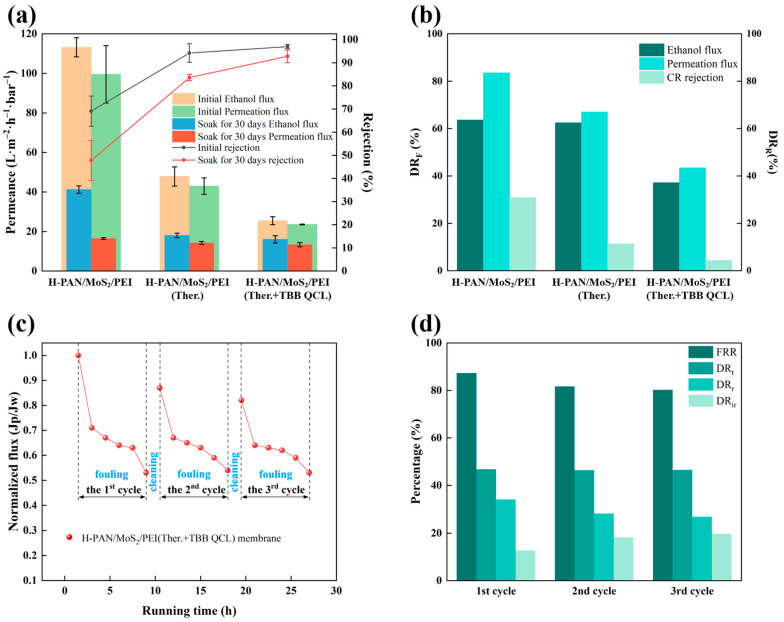
(**a**) Solvent resistance test and (**b**) performance decay rate of three membranes in ethanol before and after 30 days (OSN conditions: 0.4 MPa, 25 °C, 0.05 g·L^−1^ CR/ethanol solution); (**c**) The normalized flux during fouling-cleaning cycles and (**d**) Fouling resistance index of the H-PAN/MoS_2_/PEI (Ther.+TBB QCL) membrane (Operating conditions: 0.4 MPa, 25 °C, 0.1 g·L^−1^ HA concentration).

To assess the solvent stability of the prepared membranes, the H-PAN/MoS_2_/PEI (Ther.+TBB QCL) membrane was immersed in ethanol at 25 °C for 30 days alongside its non-crosslinked (H-PAN/MoS_2_/PEI) and thermally crosslinked (H-PAN/MoS_2_/PEI (Ther.)) counterparts, with subsequent measurement of ethanol flux, ethanol permeation flux, and Congo red rejection. As shown in Figure 6a, the pristine H-PAN/MoS_2_/PEI (Ther.+TBB QCL) membrane exhibited: Congo red rejection of 97.0 ± 0.9%, ethanol flux of 25.5 ± 2.1 L·m^−2^·h^−1^·bar^−1^, and ethanol permeation flux of 23.6 ± 0.2 L·m^−2^·h^−1^·bar^−1^. As shown in Figure 6b, following 30-day immersion, this membrane showed significantly the lowest performance reductions than the other membranes: 37.1% in ethanol flux, 43.4% in ethanol permeation flux, and only 4.3% in Congo red rejection. Those results effectively demonstrate that the dual-crosslinking approach significantly enhances membrane stability, and the crosslinked network structure restricts chain mobility, thereby effectively preventing the solvent-induced plasticization effect when compared to the non-crosslinked membranes.

During the continuous operation conditions, the membranes are subject to concentration polarization and pollutant deposition, leading to membrane fouling and flux decline. The antifouling performance of membranes is primarily characterized by flux recovery ratio (FRR). Therefore, the antifouling effect of the H-PAN/MoS_2_/PEI (Ther.+TBB QCL) membrane was investigated using the HA solution with ethanol solvent. As shown in Figure 6d, the total flux decline ratio (DR_t_) for the HA ethanol solution was 46.8%. The FRR was 87.3% in the first cycle and 80.3% after the third cycle. These results indicate that the H-PAN/MoS_2_/PEI (Ther.+TBB QCL) membrane exhibits relatively favorable antifouling properties against HA in ethanol solutions.

## 4. Conclusions

In this study, we fabricated composite membranes using hydrolyzed H-PAN as the polymeric matrix and two-dimensional MoS_2_ nanosheets as the nanofiller to prepare the casting solution. The H-PAN/MoS_2_/PEI base membranes were subsequently fabricated through a polyion complexation-assisted non-solvent-induced phase separation (PIC-NIPS) process, with PEI aqueous solution serving as the coagulation bath. The as-prepared membranes were then subjected to a dual-crosslinking process involving aqueous thermal treatment followed by 1,3,5-tris(bromomethyl)benzene (TBB) quaternization crosslinking, yielding the final H-PAN/MoS_2_/PEI (Ther.+TBB QCL) membranes for organic dye separation in ethanol systems. SEM analysis demonstrated that the selective skin layer thickness decreased from 64 nm in the pristine membrane to 50 nm after aqueous thermal treatment, and further reduced to 41 nm following quaternization crosslinking, confirming that the crosslinking process effectively enhanced the compactness of the separation layer. The H-PAN/MoS_2_/PEI (Ther.+TBB QCL) membrane exhibited exceptional separation performance in ethanol systems, achieving over 97% rejection for anionic dyes (including Congo Red and Evans Blue) with a permeance of 23.6 ± 0.2 L·m^−2^·h^−1^·bar^−1^ at 0.4 MPa operating pressure. The successful integration of dual-crosslinking methodology not only preserves the ultrathin nature of the H-PAN selective layer but also significantly enhances its ethanol stability. These findings establish a new paradigm for designing high-performance OSN membranes with both superior separation efficiency and exceptional solvent resistance, opening promising avenues for industrial organic solvent recovery and purification applications.

## Figures and Tables

**Table 1:** Comparisons of membrane performance with that of recently reported membranes.

Membrane	Ethanol Permeability (L·m^−2^·h^−1^·bar^−1^)	The Rejection of Dye (%)	Operating Condition	Reference
PDA-PA/PTFE composite nanofiltration membrane (MI-3)	5.95	CR: 86.6	cross-flow (2 bar)	[41]
PBI-LIG (1,1) membrane	12.14	CR: 94	cross-flow (5 bar)	[42]
Thin-film composite molecularly porous hyper-crosslinked membrane (TFC-MPCM)	1.69	CR: 94	cross-flow (4 bar)	[43]
SBI-OPASS TFC membrane (Mem-NaOH-4)	21.5	CR: 94.7	cross-flow (1 bar)	[44]
PIP-TMC polyamide thin-film composite membrane fabricated with 2-methyltetrahydrofuran (2-MeTHF) as organic solvent	9.87	RB: 97.1	cross-flow (3 bar)	[45]
PA/PDA-HKUST-1_0.6_/PEI membrane	31.2	CR: 92.8	cross-flow (6 bar)	[46]
PVDF/t-Cu-TCPP-(3)/PA membrane	2.75	CR: 95	cross-flow (7 bar)	[47]
Annealed crosslinked mixed matrix membrane comprising 0.05 wt.% MWCNT-COOH in P84 polyimide (M3–30)	9.6	RB: 85	cross-flow (5 bar)	[48]
H-PAN/MoS_2_/PEI (Ther.+TBB QCL) membrane	23.6	CR: 97	cross-flow (4 bar)	This work

## Data Availability

The data presented in this study are available on request from the corresponding author.

## Appendix A

**Table A1:** The properties of organic dyes.

Name	MW(Da)/Charge	Chemical Structure	3D Structure
Sunset Yellow (SY)	452/2		
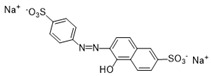
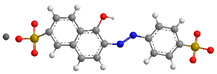
Congo Red (CR)	696/2		
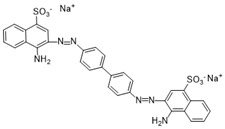
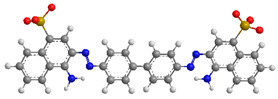
Evans Blue (EB)	961/4		
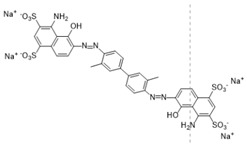
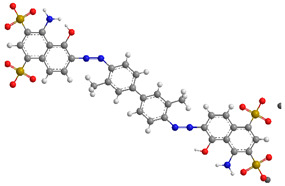
Reactive black (RB)	992/4		

## References

[1] White L.S. (2002). Transport properties of a polyimide solvent resistant nanofiltration membrane. J. Membr. Sci..

[2] White L.S., Wildemuth C.R. (2006). Aromatics Enrichment in Refinery Streams Using Hyperfiltration. Ind. Eng. Chem. Res..

[3] Kim J.F., Szekely G., Schaepertoens M., Valtcheva I.B., Jimenez-Solomon M.F., Livingston A.G. (2014). In Situ Solvent Recovery by Organic Solvent Nanofiltration. ACS Sustain. Chem. Eng..

[4] Mertens M., Van Goethem C., Thijs M., Koeckelberghs G., Vankelecom I.F.J. (2018). Crosslinked PVDF-membranes for solvent resistant nanofiltration. J. Membr. Sci..

[5] Marchetti P., Butté A., Livingston A.G. (2013). NF in organic solvent/water mixtures: Role of preferential solvation. J. Membr. Sci..

[6] Loh X.X., Sairam M., Bismarck A., Steinke J.H.G., Livingston A.G., Li K. (2009). Crosslinked integrally skinned asymmetric polyaniline membranes for use in organic solvents. J. Membr. Sci..

[7] Cook M., Gaffney P.R.J., Peeva L.G., Livingston A.G. (2018). Roll-to-roll dip coating of three different PIMs for Organic Solvent Nanofiltration. J. Membr. Sci..

[8] Marchetti P., Livingston A.G. (2015). Predictive membrane transport models for Organic Solvent Nanofiltration: How complex do we need to be?. J. Membr. Sci..

[9] Francis V.N., Chong J.Y., Yang G., Che L., Wang R. (2023). Robust polyamide-PTFE hollow fibre membranes for harsh organic solvent nanofiltration. Chem. Eng. J..

[10] Xu Y., You F., Sun H., Shao L. (2017). Realizing Mussel-Inspired Polydopamine Selective Layer with Strong Solvent Resistance in Nanofiltration toward Sustainable Reclamation. ACS Sustain. Chem. Eng..

[11] Xu Y.C., Cheng X.Q., Long J., Shao L. (2016). A novel monoamine modification strategy toward high-performance organic solvent nanofiltration (OSN) membrane for sustainable molecular separations. J. Membr. Sci..

[12] Oxley A., Livingston A.G. (2022). Anti-fouling membranes for organic solvent nanofiltration (OSN) and organic solvent ultrafiltration (OSU): Graft modified polybenzimidazole (PBI). J. Membr. Sci..

[13] Lim S.K., Goh K., Bae T.-H., Wang R. (2017). Polymer-based membranes for solvent-resistant nanofiltration: A review. Chin. J. Chem. Eng..

[14] Vanherck K., Koeckelberghs G., Vankelecom I.F.J. (2013). Crosslinking polyimides for membrane applications: A review. Prog. Polym. Sci..

[15] Sairam M., Loh X.X., Li K., Bismarck A., Steinke J.H.G., Livingston A.G. (2009). Nanoporous asymmetric polyaniline films for filtration of organic solvents. J. Membr. Sci..

[16] Sairam M., Loh X.X., Bhole Y., Sereewatthanawut I., Li K., Bismarck A., Steinke J.H.G., Livingston A.G. (2010). Spiral-wound polyaniline membrane modules for organic solvent nanofiltration (OSN). J. Membr. Sci..

[17] Zhang Y., Wang H., Li L., Zhang X., Dong X., Wang X., Pan Y., Wang T. (2024). Effects of polyacrylonitrile carboxylation on pore structure and performance of thermally crosslinked organic solvent nanofiltration membrane. Chem. Eng. Sci..

[18] Chisca S., Musteata V.-E., Zhang W., Vasylevskyi S., Falca G., Abou-Hamad E., Emwas A.-H., Altunkaya M., Nunes S.P. (2022). Polytriazole membranes with ultrathin tunable selective layer for crude oil fractionation. Science.

[19] Feng W., Li J., Fang C., Zhang L., Zhu L. (2022). Controllable thermal annealing of polyimide membranes for highly-precise organic solvent nanofiltration. J. Membr. Sci..

[20] Bhuwania N., Labreche Y., Achoundong C.S.K., Baltazar J., Burgess S.K., Karwa S., Xu L., Henderson C.L., Williams P.J., Koros W.J. (2014). Engineering substructure morphology of asymmetric carbon molecular sieve hollow fiber membranes. Carbon.

[21] Zhang C., Zhang K., Cao Y., Koros W.J. (2018). Composite Carbon Molecular Sieve Hollow Fiber Membranes: Resisting Support Densification via Silica Particle Stabilization. Ind. Eng. Chem. Res..

[22] Koh D.-Y., McCool B.A., Deckman H.W., Lively R.P. (2016). Reverse osmosis molecular differentiation of organic liquids using carbon molecular sieve membranes. Science.

[23] Han Y., Jiang Z., Gao C. (2015). High-flux graphene oxide nanofiltration membrane intercalated by carbon nanotubes. ACS Appl. Mater. Interfaces.

[24] Huang H., Song Z., Wei N., Shi L., Mao Y., Ying Y., Sun L., Xu Z., Peng X. (2013). Ultrafast viscous water flow through nanostrand-channelled graphene oxide membranes. Nat. Commun..

[25] Huang I., Ding L., Caro J., Wang H. (2023). MXene-based membranes for drinking water production. Angew. Chem. Int. Ed..

[26] Karahan H.E., Goh K., Zhang C., Yang E., Yildirim C., Chuah C.Y., Ahunbay M.G., Lee J., Tantekin-Ersolmaz S.B., Chen Y. (2020). MXene materials for designing advanced separation membranes. Adv. Mater..

[27] Xue Q., Lim Y.J., Zhang K. (2025). Engineering multi-channel water transport in surface-porous MXene nanosheets for high-performance thin-film nanocomposite membranes. J. Membr. Sci..

[28] Wang Z.-Y., Feng R., Wang W.-J., Sun Y.-X., Tao S.-N., Wang Y.-M., Chen Y.-F., Fu Z.-J., Cao X.-L., Sun S.-P. (2021). Robust braid reinforced hollow fiber membranes for organic solvent nanofiltration (OSN). Adv. Membr..

[29] Wang L., Zhang M., Shu Y., Han Q., Long L., Chen B., Wang M., Li L., Cao S., Yang Z. (2024). Covalently modified MoS_2_ for the fabrication of interlayered thin film composite membranes with excellent structural stability against swelling and drying in organic solvent nanofiltration. J. Membr. Sci..

[30] Liu J., Zhao Z., Li L., Wu Y., He H. (2023). Molecular simulation study of 2D MXene membranes for organic solvent nanofiltration. J. Membr. Sci..

[31] Wu M., Fu X., Li J., Zhao W., Li X. (2024). SWCNTs-channeled MOF nanosheet membrane for high-efficient organic solvent nanofiltration. Sep. Purif. Technol..

[32] Kandambeth S., Dey K., Banerjee R. (2019). Covalent organic frameworks for membrane separation. Chem. Soc. Rev..

[33] Wang Y., Li Z., Wang Y., Zhou Y., Zhang Y., Wang H. (2021). Two-dimensional covalent organic framework membranes for liquid molecular separation. Sci. China Mater..

[34] Bertolazzi S., Brivio J., Kis A. (2011). Stretching and Breaking of Ultrathin MoS_2_. ACS Nano.

[35] Hirunpinyopas W., Prestat E., Worrall S.D., Haigh S.J., Dryfe R.A.W., Bissett M.A. (2017). Desalination and Nanofiltration through Functionalized Laminar MoS_2_ Membranes. ACS Nano.

[36] Du J.R., Du F., Yang K., Zheng J., Ma K., Du C., Yang H. (2023). Screening of high-efficiency crosslinking agents for poly(N,N-dimethylaminoethyl methacrylate) and performance of its nanofiltration membranes. J. Membr. Sci..

[37] Naderi A., Asadi Tashvigh A., Chung T.-S. (2019). H2/CO2 separation enhancement via chemical modification of polybenzimidazole nanostructure. J. Membr. Sci..

[38] Liu C., Zhu L., Zhang Y., Hu W., Shen Y., Jin H., Wang H., Niu W. (2025). Facile fabrication of high-performance membrane with 2D GO nanosheet in membrane skin by PIC-assisted NIPS method for dye/salt separation. J. Membr. Sci..

[39] Guo H., Ma Y., Qin Z., Gu Z., Cui S., Zhang G. (2016). One-Step Transformation from Hierarchical-Structured Superhydrophilic NF Membrane into Superhydrophobic OSN Membrane with Improved Antifouling Effect. ACS Appl. Mater. Interfaces.

[40] Zhao Z., Di N., Zha Z., Wang J., Wang Z., Zhao S. (2023). Positively Charged Polyamine Nanofiltration Membrane for Precise Ion–Ion Separation. ACS Appl. Mater. Interfaces.

[41] Qiu F., Sun Y., Zhang Y., Liu H., Shao L., Huang Q. (2023). Electrospun PTFE nanofibrous composite membranes featuring a fiber network structure for organic solvent nanofiltration (OSN). Sep. Purif. Technol..

[42] Kim S.H., Khan M.A., Im K.S., Kang P., Nam S.Y. (2024). Enhanced Organic Solvent Nanofiltration Membranes with Double Permeance via Laser-Induced Graphitization of Polybenzimidazole. Adv. Mater. Interfaces.

[43] Abdul W., Umair B., Isam H.A. (2021). Fabrication of molecularly porous hyper-cross-linked thin film composite nanofiltration membrane using cyclic amine and linear cross-linker for highly selective organic solvent nanofiltration. Colloid Interface Sci. Commun..

[44] Yan X., Wan H., Xing X., Yang J., Yan G., Zhang G. (2023). High permeance nanofiltration membrane for harsh organic solvent based on spiral-ring polyesters. J. Membr. Sci..

[45] Lin S., Semiao A.C., Zhang Y., Lu S., Lau C.H. (2023). Interfacial polymerization using biobased solvents and their application as desalination and organic solvent nanofiltration membranes. J. Membr. Sci..

[46] Li H., Li X., Ouyang G., Huang L., Li L., Li W., Huang W., Li D. (2023). Ultrathin organic solvent nanofiltration membrane with polydopamine-HKUST-1 interlayer for organic solvent separation. J. Environ. Sci..

[47] Yao A., Hua D., Hong Y., Pan J., Cheng X., Tan K.B., Zhan G. (2022). Using Cu-TCPP Nanosheets as Interlayers for High-Performance Organic Solvent Nanofiltration Membranes. ACS Appl. Nano Mater..

[48] Davood Abadi Farahani M.H., Hua D., Chung T.-S. (2017). Cross-linked mixed matrix membranes consisting of carboxyl-functionalized multi-walled carbon nanotubes and P84 polyimide for organic solvent nanofiltration (OSN). Sep. Purif. Technol..

